# SWI/SNF Chromatin Remodeling Enzymes in Melanoma

**DOI:** 10.3390/epigenomes6010010

**Published:** 2022-03-18

**Authors:** Megan R. Dreier, Ivana L. de la Serna

**Affiliations:** Department of Cancer Biology, University of Toledo College of Medicine and Life Sciences, 3035 Arlington Ave, Toledo, OH 43614, USA; megan.dreier@utoledo.edu

**Keywords:** SWI/SNF enzymes, chromatin remodeling, melanocytes, melanoma, BAF, PBAF, ncBAF, epigenetics, therapeutics

## Abstract

Melanoma is an aggressive malignancy that arises from the transformation of melanocytes on the skin, mucosal membranes, and uvea of the eye. SWI/SNF chromatin remodeling enzymes are multi-subunit complexes that play important roles in the development of the melanocyte lineage and in the response to ultraviolet radiation, a key environmental risk factor for developing cutaneous melanoma. Exome sequencing has revealed frequent loss of function mutations in genes encoding SWI/SNF subunits in melanoma. However, some SWI/SNF subunits have also been demonstrated to have pro-tumorigenic roles in melanoma and to affect sensitivity to therapeutics. This review summarizes studies that have implicated SWI/SNF components in melanomagenesis and have evaluated how SWI/SNF subunits modulate the response to current therapeutics.

## 1. Introduction

### 1.1. SWI/SNF Chromatin Remodeling Complexes

Epigenetics is defined as a process that alters gene activity without changing the DNA sequence and leads to modifications that can be transmitted to daughter cells [1]. This includes DNA methylation, chromatin structure, and non-coding RNAs. Chromatin structure is regulated by two classes of enzymes: those that add or remove covalent modifications on histone proteins and ATP-dependent chromatin remodelers which utilize energy from the hydrolysis of ATP to relax contacts between histone proteins and DNA, promoting altered nucleosome conformation, positioning, or changes in higher order chromatin structure [2].

**Figure 1:**
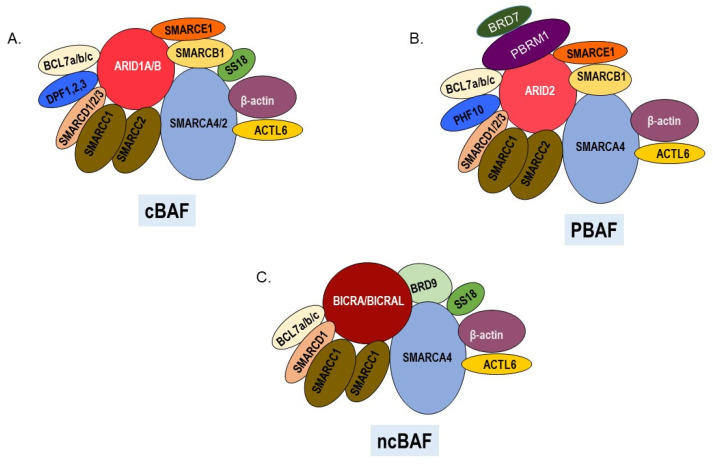
SWI/SNF chromatin remodeling complexes. All three complexes have a central ATPase, various common and unique subunits. (**A**). Canonical cBAF contains ARID1A or ARID1B and DPF1, 2, or 3 as signature subunits. The ATPase can be either SMARCA4 or SMARCA2. (**B**) PBAF complexes contain ARID2, PBRM1, BRD7, and PHF10 as signature subunits and SMARCA4 as the ATPase. (**C**) Noncanonical ncBAF contains BRD9 and BICRA/BICRAL as signature subunits and SMARCA4 as the ATPase.

SWItch/Sucrose NonFermentable (SWI/SNF), the first ATP-dependent chromatin remodeler to be identified [3], has important roles in transcription, DNA replication, and repair. SWI/SNF complexes are composed of a central catalytic subunit which is either SMARCA4 (BRG1) or SMARCA2 (BRM) and 10–13 associated subunits [4]. Heterogeneous complexes that have either the SMARCA4 or SMARCA2 catalytic subunit and a distinct assortment of accessory subunits have been labeled as canonical(c)BAF, PBAF (Polybromo-associated BAF), or noncanonical (nc)BAF [5,6] (Figure 1). Although we still do not completely understand the distinct biological roles of these diverse complexes, most SWI/SNF subunits are essential for organismal development because their deletion in mice is lethal during embryogenesis [7]. In humans, heterozygous germline mutations in genes encoding SWI/SNF components are associated with the developmental disorders including Coffin–Siris and Nicolaides–Baraitser syndromes [8]. Germline mutations in SWI/SNF genes have also been reported in patients with pediatric rhabdoid tumors [9], small cell carcinoma of the ovary, hypercalcemic type [10], and clear cell renal carcinoma [11] while somatic mutations in SWI/SNF genes occur in a diverse array of human malignancies including endometrioid carcinomas [12], intrahepatic cholangiocarcinomas [13], hepatocellular carcinoma [14], pancreatic ductal carcinoma [15], and melanoma [16,17].

Structural and biochemical studies have given insight into how SWI/SNF is assembled and how it contacts nucleosomes to remodel chromatin. While both the SMARCA4 and SMARCA2 ATPases are capable of remodeling nucleosomes in the absence of other subunits in vitro, a core complex which also contains SMARCB1, SMARCC1, and SMARCC2 has optimum chromatin remodeling activity on assembled nucleosomes [18]. High resolution cryo-electron microscopy studies recently showed that SMARCB1 interacts with the H2A/H2B acidic patch of the nucleosome, while SMARCC1/2 provides a scaffold needed for proper SWI/SNF assembly. ARID1A plays an important role in determining SWI/SNF architecture and ability to mobilize nucleosomes [19,20,21]. Characterization of the functional roles of SWI/SNF subunits has provided insight into how disruptions of particular components alter SWI/SNF activity in malignancies and other diseases.

A function of many of the SWI/SNF subunits is to promote localization of SWI/SNF complexes to specific genomic sites. For example, the importance of the ARIDs in regulating the genome occupancy of SWI/SNF is evidenced by studies showing that loss of ARID1A in neuroblastoma leads to altered localization of both cBAF and PBAF complexes [22,23]. While haploinsufficiency of ARID1A can drive cancer formation, upon its loss, the paralogous subunit, ARID1B, promotes cBAF binding to pro-tumorigenic loci [24]. Thus, disruption of ARID subunits in cancer can contribute to dysregulation of transcriptional programs, by altering SWI/SNF recruitment throughout the genome to enable tumorigenesis. Although ARIDs can facilitate non-specific binding to DNA [25], a primary mechanism for SWI/SNF recruitment to specific genome sites is through interactions with gene-specific transcriptional regulators, which can be mediated by ARIDs as well as other SWI/SNF subunits. Transcriptional regulators shown to interact with SWI/SNF include pluripotency factors [7,26], lineage-specific regulators [27,28], nuclear hormone receptors [29,30,31], tumor suppressors such as p53 [32], and oncogenes such as MYC [33]. Thus, SWI/SNF has important roles in regulating expression of genes needed for organismal development, cell differentiation, and cancer-relevant processes.

In addition to interactions with transcriptional regulators, functional domains within some of the subunits promote SWI/SNF association with chromatin by reading the epigenetic landscape. SMARCA4, SMARCA2, PBRM1, BRD7, and BRD9 have bromodomains which are evolutionarily conserved 110 amino acid modules that bind acetyl-lysines [34]. Bromodomain-containing subunits of the SWI/SNF complex have been shown to target SWI/SNF complexes to specific genes in both normal and cancer cells [35,36]. Small molecules that selectively bind the BRD9/BRD7 bromodomains are being explored as potential anticancer agents. These drugs can disrupt BRD9/BRD7 interactions with acetylated histones, and presumably promote re-localization of the SWI/SNF complex [37,38,39].

SWI/SNF enzymes operate within a network of transcription and epigenetic factors to alter the accessibility of chromatin and regulate gene expression. Early studies showed that SWI/SNF can enhance binding of transcriptional regulators by making chromatin accessible [40,41]. SWI/SNF-mediated genome-wide changes in nucleosome positioning and cooperativity or antagonism with other epigenetic regulators has been the focus of more recent studies [42,43]. SWI/SNF subunits can cooperate with other epigenetic regulators such as the BET-bromodomain family member, BRD4, as well as enhancer RNAs, to increase to activate enhancers in colorectal cancer cells [44]. SMARCA4 also interacts with BRD4 to promote MYC-driven transcriptional programs in myeloid malignancies and BRD9 interacts with BRD4 to facilitate ncBAF recruitment to chromatin in embryonic stem cells [35,42]. Therefore, the pathways that SWI/SNF regulates in a particular cell are likely to be influenced by the activities of transcription and epigenetic factors.

There is an antagonistic relationship between SWI/SNF and Polycomb Repressor Complexes (PRC) in the regulation of gene expression. PRC can block SWI/SNF-mediated chromatin remodeling [45] and both SMARCA4 and SMARCB1 can prevent EZH2, the catalytic subunit of PRC2, from binding to target promoters [46,47]. EZH2 inhibitors have recently made it to the clinic for use in some SMARCB1-deficient cancers and are being explored in a wide range of other cancers [48]. The antagonistic relationship between SWI/SNF and EZH2, and possibly other transcription and epigenetic factors, may potentially be exploited therapeutically in SWI/SNF disrupted melanoma. In this review we discuss what is known about SWI/SNF function and dysfunction in melanoma and the therapeutic implications.

### 1.2. Melanoma

Metastatic melanoma is a highly aggressive malignancy that responds poorly to chemotherapeutics and has been increasing in incidence for several decades. It was estimated that in 2020, almost 325,000 new melanoma cases were diagnosed with more than 57,000 associated deaths occurring world-wide and that, by 2040, the incidence is predicted to be 510,000 new cases with 96,000 associated deaths [49]. While targeted therapy against oncogenic mutations and immune checkpoint inhibitors have resulted in remarkable improvement in patient outcome, many patients do not respond to either therapy or they develop resistance and ultimately stop responding to the drugs [50,51,52]. Thus, it is necessary to better predict which tumors will respond to current therapies and to identify new therapeutic targets for patients who develop resistance.

Melanoma arises from the malignant transformation of melanocytes, cells responsible for melanin production. Melanin gives skin its characteristic pigmentation and protects from the damaging effects of ultraviolet (UVR) radiation coming primarily from the sun. Although melanin has a protective role, inherent features of the melanocyte differentiation program are also thought to be involved in melanoma aggressiveness and resistance to therapeutics [53,54]. The Melanocyte Inducing Transcription Factor (MITF) is a lineage-specific factor that specifies and promotes melanocyte differentiation and survival [55]. This pro-survival function carries over into melanoma, where *MITF* is a lineage addiction oncogene that is amplified in 10% of primary and more than 20% of metastatic melanoma tumors [56]. Another lineage-specific transcription factor, SRY-BOX 10 (SOX10), directly regulates MITF expression and synergizes with MITF to regulate genes encoding melanogenic enzymes, Tyrosinase (*TYR*), Tyrosinase Related Protein 1 (*TYRP1*), and Dopachrome Tautomerase (*DCT*) [57]. SOX10 also plays a role in melanomagenesis and modulates resistance to therapeutics [58,59]. Therefore, MITF and SOX10 are melanocyte-specific transcription factors that regulate pigmentation and contribute to protection against UV radiation, but also play significant roles in promoting aspects of melanoma tumorigenicity.

Exposure to UVR is a major environmental risk factor and is associated with characteristic mutational signatures for melanoma development [60]. Mutations in the *BRAF* gene occur most commonly in melanocytes in sun-exposed skin and account for approximately half of all melanomas [61]. However, mutant BRAFV600E (valine to glutamic acid substitution) in the absence of other disruptions causes senescence, giving rise to benign nevi (moles) [62,63]. The *BRAF* oncogene promotes melanoma proliferation and metastasis by hyper-activating the ERK/MEK Mitogen-Activated Pathway (MAPK) [64]. Patients with these melanomas respond well to combined BRAF/MEK inhibitors, but at least half the patients develop resistance within a year [65]. Therefore, novel approaches are needed to combat the occurrence of resistance. *NRAS* is another commonly mutated gene, accounting for approximately 20% of cutaneous melanomas, more frequently occurring on unexposed skin and associated with more aggressive disease. Treatment with MEK or immune checkpoint inhibitors has demonstrated limited efficacy for *NRAS*-mutant melanomas and there is a need to develop more effective approaches [66]. Other frequent mutations have been detected in genes encoding NF1, KIT, CDKN2A, TP53, PTEN, as well as components of the SWI/SNF complex. Mutations in genes encoding SWI/SNF subunits occur in approximately 34% of melanomas and may present vulnerabilities that can be exploited therapeutically [67].

## 2. SWI/SNF in Melanoma

### 2.1. SMARCA4

**Figure 2:**
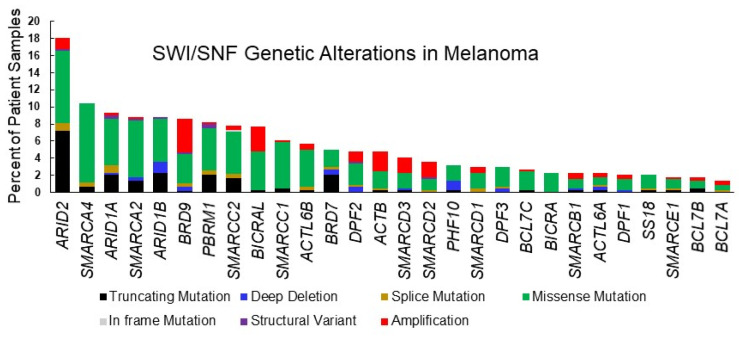
SWI/SNF subunit alterations in cutaneous melanoma. Graphic depiction of TCGA Pan Cancer Atlas datasets for cutaneous melanoma analyzed using the cBioportal tool [71,72] as in Table 1.

**Figure 3:**
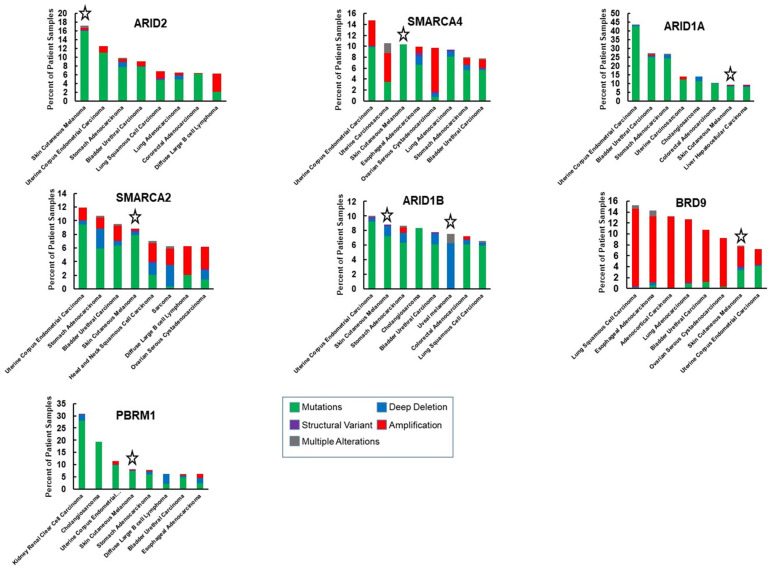
Comparison of the frequency of SWI/SNF genetic alterations in melanoma with other cancers. The graphs show seven subunits that are most frequently altered in melanoma in relation to the seven cancer types (out of 32), displaying the most frequent alterations in the respective genes. The dataset is from TCGA and evaluated with the cBioportal tool [71,72]. Stars highlight melanoma data.

*SMARCA4*, essential for mouse development [68] and embryonic stem cell pluripotency [26], has also been recognized as a tumor suppressor that is deficient in some cancers including small cell carcinoma of the ovary hypercalcemic type [10] and non-small cell lung cancer [69], but also found to be highly expressed and to have oncogenic roles in other cancers, including breast cancer and acute myeloid leukemia [42,70]. Exome studies of patient-derived melanomas have revealed mutations in *SMARCA4*, some of which are predicted to cause loss of function [16] (Table 1, Figure 2). Melanoma ranks third in its frequency of *SMARCA4* genetic alterations and while several cancers have amplifications in the *SMARCA4* gene, we did not find amplifications in the TCGA cutaneous melanoma dataset (Figure 3). Thus, there is evidence that *SMARCA4* is disrupted in melanoma, such as it is in several other cancers.

In addition to mutations, there have been variable reports of SMARCA4 protein expression in patient-derived melanoma tumors. One study found that, SMARCA4 was deficient in a significant number of primary and metastatic melanomas [73]. However, a different study showed that SMARCA4 is over expressed at the protein level in both primary and metastatic melanomas compared to nevi [74]. Other studies that have looked at SMARCA4 mRNA levels have found that SMARCA4 is highly expressed especially in later stages of metastatic melanoma and that high expression is correlated with poorer survival [75,76]. Thus, SMARCA4 status may be heterogeneous in melanoma, with some tumors exhibiting SMARCA4 loss of function while in other contexts, high levels of SMARCA4 may promote tumorigenesis.

The first report to characterize SMARCA4 function in the melanocyte lineage was one that established SMARCA4 as an MITF coactivator [28]. In this study, dominant negative SMARCA4 was found to inhibit trans-differentiation of fibroblasts to melanocyte-like cells, interfering with activation of melanogenic enzyme genes. MITF and SMARCA4 were found to physically interact and MITF recruited SMARCA4 to a melanocyte-specific promoter where it remodeled chromatin [28]. SMARCA4 was found to regulate MITF expression and to interact with MITF in melanoma cells, thereby promoting expression of an extensive number of pigmentation genes [77,78,79]. In mice, inactivation of *SMARCA4* disrupts embryonic development of the melanocyte lineage and results in a severe pigmentation phenotype [79,80]. These studies show that SMARCA4 is an essential regulator of pigmentation and survival of the melanocyte lineage as well as melanoma cells.

Multiple melanocyte-specific transcription factors likely regulate SMARCA4 recruitment to genomic sites. A genome-wide study in melanoma cells confirmed that SMARCA4 cooperates with MITF to remodel chromatin at pigmentation loci and other MITF-target genes [79]. This study indicated a high level of MITF and SMARCA4 co-localization at MITF binding sites within both proximal promoters and enhancers. These sites were configured by MITF binding to nucleosome depleted regions, flanked on both sites by SMARCA4-bound nucleosomes. Many of the MITF/SMARCA4 co-occupied sites at distal enhancers were co-occupied by SOX10 and overlapped sites for transcription factors, YY1 [81] and TFAP2A [82]. While MITF and/or SOX10 are required to recruit SMARCA4 to MITF/SOX10-dependent loci, SMARCA4 was not required for MITF to bind cognate sites. A recent report showed that MITF binding depends instead on TFAP2A, which may act as a pioneer transcription factor by binding nucleosomal DNA and promoting chromatin accessibility [83]. This suggests that SWI/SNF recruitment to MITF/TFAP2A-dependent loci indirectly depends on TFAP2A. TFAP2A expression is frequently down-regulated in advanced melanoma [84]; thus, although advanced melanomas may have high levels of SMARCA4, it is conceivable that these melanomas may become de-differentiated and invasive due to failure to target SMARCA4 and the SWI/SNF complex to differentiation-specific loci that are regulated by TFAP2A and MITF. Thus, the repertoire of transcription factors present in melanoma may dictate whether SMARCA4 has tumor-suppressive or oncogenic roles by regulating SMARCA4 genomic localization.

The role of SMARCA4 in promoting melanin synthesis likely provides protection against damage from UVR and could be considered a tumor suppressor role. SMARCA4 can also promote nucleotide excision repair and thus prevent DNA damage and accumulation of mutations [85]. Thus, disruption of SMARCA4 function is likely to contribute to the initiation of melanoma through multiple mechanisms. However, some studies have shown that SMARCA4 may also promote melanoma proliferation, invasiveness, and response to therapeutics.

SMARCA4 increases resistance to DNA-damaging agents in melanoma [77]. The increase in resistance to cisplatin and as well as survival from ultraviolet radiation-induced damage was associated with activation of *BIRC7* (ML-IAP), an MITF-regulated pro-survival gene [47]. Other studies showed that SMARCA4 is critically required for an extensive number of MITF as well as MITF-targets that are pro-survival genes in melanoma and for melanoma survival in vitro [78,79]. SMARCA4 also cooperates with an MITF-target and SCF ubiquitin ligase component, FBX032, to promote expression of genes involved in proliferation and migration [86]. MITF-independent mechanisms by which SMARCA4 promotes melanoma survival and invasiveness have also been reported [75,87,88]. Furthermore, inactivation of *Smarca4* delays tumor formation in a mouse melanoma model driven by oncogenic BRAF/inactivated PTEN [89]. In contrast to these studies which show a pro-proliferative and pro-invasive role for SMARCA4, one recent study reported that depletion of SMARCA4 transforms immortalized mouse melanocytes, allowing them to generate highly pigmented tumors in vivo [90]. Additional studies will be required to determine the basis for the ambivalent roles SMARCA4 plays in melanomagenesis as well as differences in reports of SMARCA4 expression levels. Possible explanations for the discrepancies may be due to different mutational contexts (i.e., *BRAF/PTEN* status) or differences in developmental stage and/or SMARCA2 status (see below) in the different studies. It is also conceivable that SMARCA4 levels change reversibly, depending on signals from the cancer microenvironment. It will be important to identify the contexts in which high or low SMARCA4 levels are advantageous to melanoma cells.

### 2.2. SMARCA2 (BRM)

*SMARCA2*, the paralog of SMARCA4, is the central ATPase in a subset of cBAF complexes. Although SMARCA2 is highly homologous to SMARCA4, unlike SMARCA4, mice with embryonic inactivation of *Smarca2* are viable and have not been reported to have a pigmentation phenotype [68,91]. The observation that inactivation of *Smarca4* in melanocytes during embryogenesis results in loss of melanocytes suggests that SMARCA2 cannot compensate for SMARCA4 during embryonic development of the melanocyte lineage [79]. Despite this hypothesis, SMARCA2 was shown to interact with MITF and to partially compensate for SMARCA4 loss in the regulation of MITF-target genes in melanoma cells [77]. A region near *SMARCA2* has also been associated with genetic variation in pigmentation across African populations [92], suggesting the possibility that SMARCA2 does have a role in the regulation of melanin synthesis.

*SMARCA2* mutations occur at almost the same frequency as SMARCA4 mutations in patient derived melanomas (Table 1, Figure 2). Melanoma ranks as the fourth most common cancer with *SMARCA2* genetic alterations (Figure 3). High frequency of *SMARCA2* mutations also occur in non-melanoma skin cancers and are associated with damage from UVR [93], suggesting that SMARCA2 has a tumor-suppressive function when skin is exposed to the sun. A tumor-suppressive function may be attributed to the association of SMARCA2 with senescence in melanocytic nevi [94]. Interestingly, mechanisms other than mutations have been shown to inactivate SMARCA2. Oncogenic RAS and RAF can epigenetically silence SMARCA2 expression and protein acetylation can suppress SMARCA2 activity [95,96,97]. These observations suggest a possible tumor-suppressive function for SMARCA2 in melanoma that should be more closely evaluated.

SMARCA2 and SMARCA4 exhibit a synthetic lethal relationship in cancer that can be exploited therapeutically. Depletion of SMARCA2 in SMARCA4-deficient melanoma cells abrogates melanoma tumorigenicity [77] and systematic studies in other cancers have shown that loss of one subunit renders cells highly dependent on the paralogous subunit [98]. SMARCA4 and SMARCA2 have homologous functional domains that can be targeted with small molecules. Dual allosteric inhibitors of the SMARCA2/SMARCA4 ATPases simulate synthetic lethality by curbing proliferation of SMARCA4-deficient cancers [99]. Furthermore, AADi, an inhibitor specific for the ATPase domains of chromatin remodeling enzymes also has anti-cancer effects [100]. While bromodomain inhibition was not as effective as ATPase inhibition at curbing growth of some SWI/SNF mutant cancers [101], PFI-3, a small molecule selective for the SMARCA4/SMARCA2/PBRM1 bromodomains, sensitized cancer cells to DNA-damaging agents [102,103,104]. Thus, there is therapeutic potential in targeting the functional domains of SMARCA2 and SMARCA4 in some cancer contexts, particularly when there is a deficiency in one ATPase. It will be important to further evaluate potential synthetic lethality between SMARCA4 and SMARCA2 in melanoma.

### 2.3. SMARCB1 (INI1/BAF47/SNF5)

SMARCB1 is a core component of cBAF and PBAF complexes which has been implicated in melanoma and other cancers. Although *SMARCB1* is not frequently mutated in melanoma (Figure 2, Table 1), inactivating mutations in *SMARCB1* frequently occur in a number of other cancers, especially in pediatric rhabdoid tumors, where there is frequent biallelic SMARCB1 disruption [105,106,107,108]. Mechanistically, cancer-associated mutations in the C-terminal domain of *SMARCB1* disrupt interactions with the nucleosome acidic patch and alter SWI/SNF binding genome-wide [109]. *Smarcb1* disruption is early embryonic lethal and mice with heterozygous disruption of *Smarcb1* develop tumors which exhibit loss of heterozygosity, indicating that SMARCB1 is a bona fide tumor suppressor [110,111]. Furthermore, SMARCB1 functions in nucleotide excision repair [112], which is critically important to prevent damage from solar UVR. Therefore, it is somewhat surprising that *SMARCB1* is not more frequently mutated in melanoma. However, *Smarcb1* is an essential gene for mouse development and a core subunit in both cBAF and PBAF complexes. While some cancer cells have bypassed this requirement, SMARCB1 may be still be essential for melanoma survival, perhaps as a consequence of its lineage-specific functions (described below). Thus, mis-expression of SMARCB1 could be a more advantageous route toward disruption of SMARCB1 function.

Immunohistochemistry on patient-derived melanoma samples indicated there is significantly lower SMARCB1 expression in late stage primary and metastatic melanoma, correlating with poorer patient survival and increased resistance to chemotherapeutics [113]. A screen identified SMARCB1 as a factor required for mutant BRAF-induced senescence, suggesting a tumor suppressor role for SMARCB1 in melanomas that harbor this oncogene [114]. However a different study challenged this finding [115] and it has recently been reported that loss of SMARCB1 results in senescence by suppressing SOX10 expression in melanoma cells [116]. The regulation of a melanocyte-specific factor like SOX10 by SMARCB1 indicates that SMARCB1 likely is essential for survival of the melanocyte lineage and in melanoma. Interestingly, SMARCB1-depleted melanoma cells were more resistant to BRAF inhibitors but more sensitive to BCL2 inhibitors, providing additional evidence that SMARCB1 expression can influence sensitivity to therapeutics.

### 2.4. SMARCD1, 2, 3 (BAF60A, B, C)

SMARCD1, 2, and 3 are paralogues that are incorporated into the SWI/SNF complex in a mutually exclusive manner and often mediate interactions between SWI/SNF and gene-specific transcriptional regulators. The genes encoding these subunits are mutated at low frequency in melanoma, with *SMARCD3* being slightly more frequently altered then the *SMARCD1* and *SMARCD2* genes (Figure 2, Table 1). *SMARCD2* and *SMARCD3* alterations include similar frequencies of mutations as amplifications while *SMARCD1* alterations are mostly mutations. The following studies have given insight into unique and overlapping functions attributed to the different SMARCD-paralogues.

SMARCD1 is unique among the SMARCD-paralogues in that it can be a component of any of the different SWI/SNF complexes (cBAF, PBAF, and ncBAF). It is also a component of a specialized embryonic stem cell SWI/SNF complex that is associated with bivalent marks and can be both an activator and repressor [117,118]. SMARCD1 mediates SWI/SNF interactions with many different transcription factors, including nuclear hormone receptors and p53 [29,32]. Although SMARCD1 has been more extensively characterized for its developmental role and interaction with transcription factors, SMARCD2 has been shown to be required for neutrophil differentiation [119,120] and to preserve cellular identity through a p53/ATM-mediated mechanism [121].

SMARCD1 and SMARCD2 both interact with MITF and may have important roles in melanocyte development and melanoma [79,122]. SMARCD1 also interacts with SOX10 in other neural crest-derived cells [123]. Depletion of SMARCD1 in differentiating melanoblasts inhibited melanin synthesis and the expression of MITF target genes [122]. Moreover, it was shown that MITF recruits SMARCD1 to melanocyte-specific promoters, and SMARCD1 is required for recruitment of SMARCA4, suggesting that SMARCD1 mediates MITF interactions with the SWI/SNF complex. SMARCD1 and SMARCD2 also co-immunoprecitated with MITF in melanoma cells; however, the role these paralogues play in regulating melanoma tumorigenicity is not known [79]. Although SMARCD2 has not been functionally characterized in the melanocyte lineage, it potentially co-activates MITF target genes. It will be interesting to determine if SMARCD1- and SMARCD2-containing complexes regulate different classes of MITF target genes or whether they are functionally redundant in this capacity. Because of their demonstrated interactions with MITF, it will be important to conduct more extensive functional studies of both SMARCD1 and SMARCD2 during melanocyte development and in melanoma.

While SMARCD3 interacts with MYOD [124] and is critical for muscle differentiation, it interacts weakly if at all with MITF in differentiating melanoblasts [122]. However, it remains possible that SMARCD3 could be important for melanocyte differentiation by interacting with other melanocyte-specific transcription factors. Furthermore, the demonstrated roles of SMARCD3 in glycolytic metabolism [125] and lipogenesis [126] could have implications for melanocyte development and melanoma proliferation. Interestingly, it was reported that high expression of SMARCD3 correlates with poorer patient survival in uveal melanoma [127]. Therefore, a thorough investigation of SMARCD3 in both cutaneous and uveal melanoma is warranted.

### 2.5. ARID1A andARID1B

*ARID1A* is the most frequently mutated SWI/SNF gene and one of ten most commonly mutated driver genes in human cancers [128]. In melanoma, *ARID1A* is the third most frequently mutated SWI/SNF, occurring in 9% of melanoma tumors within the TCGA database (Table 1, Figure 2). Cutaneous melanoma ranks 7 among 32 different human cancers in the observed frequency of genetic alterations in *ARID1A* (Figure 3). A study utilizing sequence capture analysis of 114 melanoma patients detected loss of function mutations in *ARID1A* which were associated with significantly reduced expression [129]. Interestingly, *ARID1A* mutations were detected more frequently on the head and extremities, compared to the trunk, and *ARID1A*-truncating mutations were associated with later stages of melanoma progression, correlating with an increase in the expression of an EZH2 transcriptional program [130]. Mutations in *ARID1A* have also been associated with melanoma metastasis to the brain [131].

ARID1A loss can have therapeutic implications by modulating the response to immunotherapy. Melanoma patients who had tumors with high levels of wild type ARID1A expression were found to have better responses to immune checkpoint inhibitors while patients with tumors having mutations in ARID1A had a poorer response [132]. Mechanistically, ARID1A was found to promote interferon γ-regulated genes by antagonizing EZH2. However, a different report suggested that other cancer types with loss of ARID1A are more sensitive to immune checkpoint blockade [133]. Tumors with loss of ARID1A were also found to be more sensitive to immune checkpoint blockade when combined with glutaminase [134] or ATM inhibition [135]. Loss of ARID1A also increased sensitivity to EZH2 [136] and BET [137] inhibitors but decreased sensitivity to mTOR inhibitors [138]. Therefore, how ARID1A affects sensitivity to immunotherapy is ambiguous and requires additional studies.

The frequency of *ARID1B* mutations in cutaneous melanoma approaches that of *ARID1A* (Table 1, Figure 2). Interestingly, both cutaneous and uveal melanomas are among the cancers with the most frequent *ARID1B* genetic alterations (Figure 3). ARID1B is approximately 50% identical at the amino acid level with ARID1A and is incorporated into cBAF complexes in a mutually exclusive manner as ARID1A [139]. A study in which targeted next generation sequencing was conducted on 38 treatment naive melanoma patients also found *ARID1B* to be mutated at the same rate as *ARID1A* (13.2%) [140]. Interestingly, mutations in both *ARID1A* and *ARID1B* were associated with a UVB-mutational signature. However, a meta-analysis of mucosal melanomas suggests that copy number losses in *ARID1B* (33.3%) occur more frequently than in *ARID1A* (8.3%) for this melanoma sub-type [141]. Mucosal melanoma is a subtype of melanoma that constitutes a greater proportion of melanoma cases in non-Europeans, developing from non-UV exposed melanocytes on mucosal surfaces such as the sinonasal tract, oral cavity, female genital tract, the anus, and urinary tracts [142]. Thus, in cutaneous, mucosal, and uveal melanomas, there is a strikingly high frequency of mutations in *ARID1B*.

Although there have not been any functional studies of *ARID1B* in melanoma, other cancer cells with mutations in *ARID1A* are highly vulnerable to *ARID1B* loss [24,143]. However, concurrent loss of *ARID1A* and *ARID1B* has also been detected in some cancers [143] and in mice, dual deletion of *Arid1a* and *Arid1b* causes de-differentiation and promotes liver, squamous cell carcinoma, and endometrial cancers [144]. In combination, these studies suggest that the outcome of dual *ARID1A/ARID1B* loss, as being either synthetic lethal or highly carcinogenic, is context dependent. We did not see frequent concurrent mutations in *ARID1A* and *ARID1B* in melanomas in the TCGA database (Table 1) (only three melanoma tumors had mutations in both *ARID1A* and *ARID1B* out of the 41 *ARID1A* mutated samples), suggesting the possibility that in many melanomas, there is a synthetic lethal relationship between the two paralogues. However, this needs further evaluation.

Several studies indicate that ARID1A/ARID1B have important roles in maintaining enhancer activity, particularly near genes involved in cell adhesion, development, and differentiation [22,24]. As of yet, there have not been any studies investigating genomic occupancy of ARID1A/B in melanocytes and melanoma nor their role in melanocyte development or differentiation. Melanocyte and melanoma differentiation is critically dependent on MITF. Although neither ARID1A nor ARID1B were identified as MITF-interacting proteins in melanoma cells, this does not preclude the possibility that ARID1A and/or ARID1B are involved in differentiation through interactions with other transcription factors, such as SOX10 and TFAP2A, which collaborate with MITF, or potentially other transcription factors that independently promote differentiation. It will be important to elucidate the functions of ARID1A and ARID1B both during melanocyte development and in melanoma models, and to elucidate how they regulate enhancer function to regulate gene expression.

### 2.6. ARID2 and Other Components of the PBAF Complex

PBAF-specific *ARID2* is the most frequently mutated SWI/SNF gene in melanoma [16,17,145] (Table 1, Figure 2). Moreover, melanoma ranks as the cancer that most frequently displays genetic alterations in *ARID2* (Figure 3). Like mutations in *ARID1A* and *ARID1B*, mutations in *ARID2* are associated with a UVB signature [140]. ARID2 has been reported to play a role in DNA repair and maintenance of genome integrity, as do several other SWI/SNF subunits [146,147]. As part of the PBAF complex, ARID2 may have a specific role in transcriptional repression at DNA damaged sites [148]. Still, it is not clear why *ARID2* is so frequently mutated in melanoma compared to other SWI/SNF subunits, including other components of PBAF like PBRM1. Components of the PBAF complex have been identified as also being components of the MITF interactome [79]. However, a functional role for ARID2 in the regulation of the MITF transcriptional program or in melanocyte development has not been determined. In one study looking at the evolution of melanoma, *ARID2* mutations were not detected in benign nevi but instead coincided with the transition to melanoma in situ, and occurred earlier than *ARID1A* and *ARID1B* mutations [130]. This study provided insight into *ARID2* disruption in melanoma, by indicating that *ARID2* is disrupted early during melanomagenesis and suggesting that it may be tied to UVR.

The prevalence of loss of function mutations in *ARID2* suggests it is a tumor suppressor in melanoma. However, *ARID2* mutations have also been detected in melanocytes from normal skin subject to high cumulative sun exposure. This suggests that loss of ARID2 function contributes to progression, but is not sufficient to transform melanocytes [149]. Functional studies suggest that ARID2 does not affect melanoma proliferation but instead suppresses melanoma invasion in vitro [150]. Consistent with a role in cancer progression, ARID2 loss was found to suppress metastasis in a mouse model of hepatocellular carcinoma [151]. Additional in vivo studies are clearly needed to elucidate the function of ARID2 in melanocyte development and in tumor suppression.

Although the tumor-suppressive functions of ARID2 in melanoma are not completely understood, loss of ARID2 has been associated with increased sensitivity to immune checkpoint inhibitors by two independent studies [150,152]. ARID2 was found to be a transcriptional repressor of STAT1 expression in melanoma cells and depletion of ARID2 enhanced the interferon γ response, resulting in increased STAT1 and STAT1 target gene expression, including PDL1 as well as several T cell chemokines. ARID2 loss resulted in greater infiltration of cytotoxic CD8+ T cells and reduced tumor burden in response to anti-PDL1 antibody in a syngeneic mouse melanoma model [150]. In patient-derived melanoma tumors, low ARID2 expression was associated with increased patient survival when tumors had greater CD8+ T cell infiltration, suggesting ARID2 loss enhances tumor immunity [152].

Other components of the PBAF complex have also been implicated in tumor immunity and sensitivity to immunotherapy. *PBRM1* and *BRD7*, which are mutated at lower frequency in melanoma than *ARID2* (Table 1, Figure 2), were also identified in an unbiased CRISPR/CAS9 screen as modulators of resistance to T cell-mediated killing and sensitivity to immunotherapy [152]. Expression of interferon γ inducible genes, including chemokines that recruit effector T cells, increased in PBRM1-depleted mouse melanoma cells and this was associated with increased chromatin accessibility at regulatory sites. In fact, there was extensive overlap in transcriptomic changes between ARID2- and PBRM1-depleted cells, suggesting coordinated regulation of gene expression by these two members of the PBAF complex.

PBRM1 is a PBAF subunit that has six tandem bromodomains and the gene is most frequently mutated in renal clear cell carcinoma [153] and at lower frequencies in other cancers, including melanoma (Table 2). Although not as frequently mutated in melanoma as *ARID2* and *SMARCA4*, PBRM1 protein levels are highly sensitive to the levels of SMARCA4 protein [47] and incorporation into the SWI/SNF complex requires the presence of ARID2 [154]. Therefore, perturbations in *SMARCA4* or *ARID2* may also disrupt PBRM1 function if the genetic alterations result in truncated proteins or loss of protein expression. As a component of the MITF interactome, PBRM1 may have a role in melanocyte development, however, this remains to be determined. While PBRM1 plays an essential role in cardiac development [155], recent studies suggest that *Pbrm1* is dispensable for skeletal muscle differentiation [36] and for Schwann development [156]. Thus, its role in lineage specification and developmental gene expression is likely to be cell specific. In addition to modulation of melanoma sensitivity to immunotherapy, a study suggests that loss of PBRM1 confers synthetic lethality to inhibitors of DNA repair [157]. Therefore, there are multiple therapeutic opportunities for exploiting PBRM1 disruptions in melanoma.

BRD7 is a bromodomain-containing subunit of PBAF that is generally regarded as a tumor suppressor due to its positive role in the regulation of p53-induced senescence [158,159,160]. However, a recent study which analyzed publicly available datasets found that BRD7 is over-expressed in melanoma and that expression increases in metastatic disease [161]. TP-472, a drug that is selective for the highly similar bromodomains of BRD7 and BRD9, markedly inhibited melanoma growth and invasion. The anti-tumor effects of TP-422 were associated with changes in the expression of extracellular matrix and apoptotic genes, suggesting that either BRD7 or BRD9 or both promote tumor growth and invasiveness. Consistent with a role for BRD7 as pro-tumorigenic in some contexts, a different study found BRD7 stabilizes MYC and promotes colorectal tumor growth [162]. BRD7 as well as ARID2 were also associated with MYC and MYC target gene expression and with poorer prognosis in multiple myeloma [163]. Therefore, there are multiple reports that challenge the role of BRD7 as a tumor suppressor.

PHF10 is a PBAF subunit with controversial roles in melanoma and other cancers. A Drosophila study suggests that PHF10 is involved in transcriptional elongation [164]. PHF10 has also been found to activate NF-kb target genes [165]. In uveal melanoma, *PHF10* is subject to homozygous deletion and frame shift mutations [166]. We found that *PHF10* is not as frequently mutated as other PBAF components in cutaneous melanoma (Table 1, Figure 2). It has been reported that PHF10 is over-expressed in cutaneous melanoma and interacts with MYC, to recruit the PBAF complex to pro-proliferative loci, and promote cell cycle progression [167]. PHF10 also had a pro-proliferative role in gastric cancer cells [168,169]. These studies on different PBAF subunits suggest that the tumor-suppressive role of PBAF is not clear cut and warrants further investigation.

**Table 2:** SWI/SNF subunit function and roles in melanoma and other cancers. A summary of studies on SWI/SNF subunits that have been implicated in melanoma which were discussed within the text.

Subunit	Function in SWI/SNF	General Cellular Functions	Specific Functions in Melanocytes/Melanoma
SMARCA4	Central ATPase in PBAF, ncBAF, and a subset of cBAF complexes; also has a bromodomain [5,6,34].	Required for mouse development [68], embryonic stem cell pluripotency [26], promotes nucleotide excision repair [85].	Has ambivalent roles with some reports indicating low expression [73] and others high expression [74,75,76]. Required for melanocyte development, melanoma tumorigenicity, co-activator for MITF and SOX10, promotes melanin synthesis, increases resistance to DNA-damaging agents [47,75,77,78,79,80]. Promotes tumorigenesis in BRAFV600E-driven mouse models [89]. Suppresses tumorigenesis in orthotopic models of melanoma [90].
SMARCA2	Central ATPase in a subset of cBAF complexes; also has a bromodomain [5,6,34].	High frequency of mutations in sun-exposed non-melanoma skin cancers [93]. Expression can be suppressed by oncogenes and activity inhibited by acetylation [95,96]. Synthetic lethal with SMARCA4 [98].	Interacts with MITF and compensates for SMARCA4 loss in some melanoma cells [77]. Associated with human variation in pigmentation [92] and with senescent melanocytes [94].
SMARCB1	Core component of cBAF and PBAF complexes. Interacts with the acidic patch of the nucleosome [18,19,20,21].	Homozygous disruption is embryonic lethal; mice with heterozygous disruption develop tumors with loss of heterozygosity [110,111]. Involved in nucleotide excision repair [112].	Has ambivalent roles. May be required for mutant BRAF-induced senescence [114]. Loss also results in senescence, increasing sensitivity to BCL2 inhibitors and resistance to BRAF inhibitors [116].
SMARCD1	Component of ncBAF and a subset of cBAF and PBAF complexes [5,6].	Associated with embryonic stem cell self-renewal and pluripotency, bivalent marks, nuclear hormone, p53, SOX10 (Schwann cell) interactions [29,32,117,118,123].	Interacts with MITF and SOX10 in melanocytes and melanoma cells [79,122].
SMARCD2	Component of a subset of cBAF and PBAF complexes [5,6]	Involved in neutrophil differentiation, interacts with p53 and ATM to preserve cell identity [119,120,121].	Interacts with MITF in melanocytes and melanoma cells [79,122].
SMARCD3	Component of a subset of cBAF and PBAF complexes [5,6].	Required for muscle differentiation [124]. Involved in glycolytic metabolism and lipogenesis [125,126].	Correlates with poorer patient survival in uveal melanoma [127].
ARID1A	Component of some cBAF complexes. Has important function in determining SWI/SNF architecture and ability to mobilize nucleosomes [19,20,21].	Most frequently mutated SWI/SNF gene in cancer [128]. Promotes expression of interferon γ-regulated genes [132]. Associated with lineage-specific enhancers [22,24].	Mutations associated with late stages and EZH2 program. Melanoma patients with tumors that have high levels correlate with better response to immune checkpoint inhibitors [132].
ARID1B	Component of a subset of cBAF complexes [5,6].	Associated with lineage-specific enhancer activation [22,24]. Compensates for ARID1A loss in some cancers and is synthetic lethal with ARID1A loss [24,143]. Dual loss of ARID1A/ARID1B can also be pro-tumorigenic [144].	High frequency of copy-number losses in mucosal melanomas [141]. High frequency of deep deletion in uveal melanoma (Figure 3).
ARID2	Component of PBAF complexes [5,6].	Functions in DNA repair and genome integrity [146,147]. Occupies and activates lineage-specific enhancers during osteogenesis [170].	Mutations are associated with UVR exposure and coincide with the transition to melanoma in situ [130]. Suppresses invasion in vitro and modulates response to immunotherapy in vivo [150,152].
PBRM1	Component of PBAF complexes that has six tandem bromodomains [5,6,34].	Frequently mutated in renal clear cell carcinoma 153]. Loss is synthetic lethal with inhibitors of DNA repair [157].	Component of MITF interactome [79]. Modulates response to immunotherapy by regulating interferon γ inducible genes [152].
BRD7	Bromodomain-containing component of PBAF complexes [5,6,34].	Positive regulator of p53-induced senescence [158,159,160]; also interacts with MYC, promotes colorectal cancer growth and is associated with poorer prognosis in multiple myeloma [162,163].	High expression was associated with poorer patient survival and anti-tumorigenic response obtained with TP-772 [161].
PHF10	Component of PBAF complexes [5,6].	In Drosophila, involved in transcriptional elongation [164]. Activates NF-kβ target genes [165]. Promotes proliferation of gastric cancer cells [168,169].	Homozygous deletion and frame-shift mutations in uveal melanoma [166]. Over-expressed in cutaneous melanoma and interacts with MYC to promote proliferation [167].
BRD9	Bromodomain-containing component of ncBAF complexes [5,6,34].	Vulnerability in cancers with SMARCB1 inactivation [171] and tumors with SS18-SSX fusion [172]. Inhibition of BRD9 suppresses tumorigenicity of diverse cancers [37,38,173,174].	Ambivalent role in melanoma. Over-expressed in melanoma and associated with the anti-tumorigenic response to TP-772 [161]. Expression is lost in uveal melanoma due to mis-splicing and incorporation of a poison exon as a result of mutations in SF3B1 [90].

### 2.7. BRD9 and ncBAF

BRD9 is a bromodomain-containing component of ncBAF, which includes SMARCA4 as the ATPase, and a unique subunit, BICRA/BICRAL (Figure 1C). *BRD9* is frequently amplified in cancer (Table 1, Figure 2) and is a vulnerability in cancer cells with inactivation of SMARCB1 [171] or tumors having an oncogenic *SS18-SSX* fusion [172]. Recurrent focal amplifications of *BRD9* have been associated with tumorigenicity in several cancers and inhibition of BRD9 was found to suppress breast, ovarian, gastrointestinal stromal cancer, and prostate tumor growth [38,173,174] as well as proliferation of acute myeloid leukemia cells [37]. *BRD9* disruptions occur in 8% of TCGA cutaneous melanoma tumors, approximately half of which are amplifications and half missense mutations (Table 1, Figure 2). Although *BRD9* amplifications occur more frequently in other cancers (Figure 3), the TCGA dataset indicates that *BRD9* is the most frequently amplified SWI/SNF gene in cutaneous melanoma, closely followed by *BICRAL1*, which encodes another ncBAF component (Table 1, Figure 2). It was recently reported that BRD9 is over-expressed in melanoma and that high expression of BRD9 correlates with poorer survival and that it may be a therapeutic target [161].

There is also evidence that BRD9 is potentially a tumor suppressor in both uveal and cutaneous melanoma and possibly other cancers. Loss of BRD9 expression was shown to occur in uveal and cutaneous melanoma cells by a mechanism involving recurrent mutations in a splicesomal factor, SF3B1 [90]. This study found that mutant SF3B1 disrupted BRD9 expression by promoting a mis-splicing event that introduces a poison exon, leading to degradation of BRD9 mRNA. Inhibition of BRD9 was associated with reduced ncBAF localization to CTCF-associated loci, suggesting a potential requirement for BRD9 in the regulation of higher order chromatin structure. Loss of BRD9 promoted melanoma proliferation in vitro and tumor growth and metastasis in vivo and correction of the poison exon in *BRD9* using antisense oligonucleotides slowed tumor growth. Thus, there is evidence that in some melanomas with SF3B1 mutations and loss of BRD9, an approach which restores BRD9 expression may potentially be therapeutically useful.

## 3. Conclusions

SWI/SNF enzymes are a multi-subunit complexes that play important roles in transcription, organismal development, cellular differentiation, and DNA repair, and are frequently perturbed in cancer. Melanoma is characterized by a high frequency of SWI/SNF mutations, many of which are predicted to cause loss of function. Tumor suppressor functions for SWI/SNF likely involve regulation of melanin synthesis and DNA repair, two important processes that protect melanocytes and other cutaneous cells from the damaging effects of UVR. Studies have also suggested that some SWI/SNF components can suppress proliferation or invasion. Although the mechanisms by which SWI/SNF exerts tumor suppression in melanoma have not been clearly delineated, they may involve antagonism with EZH2, a validated oncogene in melanoma [175,176]. Table 2 highlights some of the properties and functions of selected SWI/SNF subunits in melanoma and other cell types which have been discussed in this review.

A remaining gap is our understanding of why mutations in genes encoding some SWI/SNF subunits, particularly *ARID2*, are over-represented in melanoma compared to mutations in genes encoding other subunits with known tumor-suppressor functions such as *SMARCB1*. It has been suggested that the special requirement for SWI/SNF complexes at lineage-specific enhancers underlies SWI/SNF-mediated tumor suppression [177]. In support of lineage-specific enhancer function, ARID2 and PBAF components, PBRM1 and BRD7, have recently been reported to occupy osteogenic gene enhancers and to promote open chromatin during osteogenesis [170]. In melanoma, SMARCA4 binds and is required for active enhancer function near melanocyte development genes [79]. The contribution of ARID2 and other PBAF subunits to the regulation of melanocyte-specific gene enhancers is suggested by the observed interactions with MITF [79], but this remains to be functionally investigated. If ARID2 is required for melanocyte-specific enhancer function, loss of function mutations in *ARID2* would likely result in melanocyte de-differentiation, a process that has been associated with susceptibility to oncogene transformation and melanoma progression [178,179]. Although SMARCA4 and SMARCB1 are also required for SWI/SNF-mediated chromatin remodeling at lineage-specific enhancers [180], loss of these subunits may be inconsistent with melanocyte and melanoma viability under most contexts due to their broader role in the regulation of gene expression. It will be important to investigate ARID2 loss during development of the melanocyte lineage in vivo and to evaluate its effects on viability in order to better understand its tumor-suppressive activities. The unique functions of ARID2 compared to the other ARIDs (ARID1A and ARID1B) will increase understanding of why *ARID2* is more frequently mutated in melanoma.

Some studies have challenged the paradigm that SWI/SNF is strictly tumor suppressive in melanoma. This is supported by the observation that SWI/SNF components can be over-expressed and interact with oncogenes to promote tumorigenesis. Moreover, mutations in SWI/SNF genes are frequently heterozygous, leaving one allele intact. Since SWI/SNF regulates many biological processes, it is likely that tumor-promoting or -suppressive roles are context dependent and possibly influenced by the microenvironment. In order to resolve ambiguous SWI/SNF functions, it will be important to elucidate the mechanisms by which individual subunits promote transcriptional programs at different stages of melanocyte development and during melanomagenesis. In this regard, more genomic studies are needed. Additional insight into SWI/SNF function in melanocytes and melanoma could be gained by elucidating the role of SWI/SNF in the regulation of higher order chromatin structure and the integration of SWI/SNF functions with the activities of additional melanocyte/melanoma specific transcription factors and other epigenetic regulators. Filling these gaps could identify steps that are amenable to drug treatment.

## Figures and Tables

**Table 1:** The first column contains the percentages of genetic alterations and, in parentheses, the total numbers of affected samples. The percentages and, in parentheses, the number of mutations/copy numbers/structural variants are shown in subsequent columns. The numbers are based on 448 samples taken from 442 melanoma patients. Subunits are listed in rank order based on number of genetic alterations. * indicate one sample with multiple genetic alterations and ** indicate two samples with additional alterations. The data was analyzed using the cBioportal tool [71,72].

Gene	Total	Truncating Mutation	Deep Deletion	Splice Mutation	Missense Mutation	In frame Mutation	Structural Variant	Amplification
*ARID2*	18%	41%	0	5%	47%	0	1%	8%
	(78)	(32) *		(4)	(37) *		(1)	(6) **
*SMARCA4*	10%	7%	0	4%	89%	0	0	0
	(46)	(3)		(2)	(41)			
*ARID1A*	9%	22%	2%	10%	59%	0	5%	2%
	(41)	(9)	(1)	(4)	(24)		(2)	(1) *
*SMARCA2*	9%	15%	5%	0	74%	0	5%	3%
	(39)	(6)	(2)		(29)		(2)	(1)
*ARID1B*	9%	26%	15%	0	56%	0	3%	0
	(39)	(10)	(6)		(22)		(1)	
*BRD9*	8%	3%	5%	5%	41%	0	3%	46%
	(37)	(1) *	(2)	(2)	(15)		(1)	(17) *
*PBRM1*	8%	36%	0	6%	61%	0	6%	3%
	(36)	(9)		(2)	(22)		(2)	(1)
*SMARCC2*	8%	21%	0	6%	65%	3%	0	6%
	(34)	(7)		(2)	(22)	(1)		(2)
*BICRAL*	8%	3%	0	0	59%	0	0	38%
	(34)	(1)			(20)			(13)
*SMARCC1*	6%	7%	0	0	89%	0	0	4%
	(27)	(2)			(24)			(1)
*ACTL6B*	6%	4%	0	8%	76%	0	0	12%
	(25)	(1)		(2)	(19)			(3)
*BRD7*	5%	41%	14%	5%	41%	0	0	0
	(22)	(9)	(3)	(1)	(9)			
*DPF2*	5%	0	14%	5%	52%	0	5%	24%
	(21)		(3)	(1)	(11)		(1)	(5)
*ACTB*	5%	5%	0	5%	43%	0	0	48%
	(21)	(1)		(1)	(9)			(10)
*SMARCD3*	4%	6%	6%	0	44%	0	0	44%
	(18)	(1)	(1)		(8)			(8)
*SMARCD2*	3%	0	0	7%	40%	0	7%	53%
	(15)			(1)	(6)		(1) *	(8) *
*PHF10*	3%	7%	36%	0	57%	0	0	0
	(14)	(1)	(5)		(8)			
*SMARCD1*	3%	0	0	15%	53%	0	0	23%
	(13)			(2)	(8)			(3)
*DPF3*	3%	0	15%	8%	77%	0	0	0
	(13)		(2)	(1)	10			
*BCL7C*	3%	8%	0	0	83%	0	0	8%
	(12)	(1)			(10)			(1)
*BICRA*	2%	0	0	0	100	0	0	0
	(10)				(10)			
*SMARCB1*	2%	10%	10%	0	50%	0	0	30%
	(10)	(1)	(1)		(5)			(3)
*ACTL6A*	2%	10%	20%	10%	40%	0	0	20%
	(10)	(1)	(2)	(1)	(4)			(2)
*DPF1*	2%	0	11%	0	67%	0	0	22%
	(9)		(1)		(6)			(2)
*SS18*	2%	11%	0	11%	78%	0	0	0
	(9)	(1)		(1)	(7)			
*SMARCE1*	2%	13%	0	13%	63%	0	0	13%
	(8)	(1)		(1)	(5)			(1)
*BCL7B*	2%	25%	0	0	50%	0	0	25%
	(8)	(2)			(4)			(2)
*BCL7A*	1%	0	0	17%	50%	0	0	33%
	(6)			(1)	(3)			(2)

## Data Availability

The data is derived from The Cancer Genome Atlats (TCGA) datasets using the cBioportal site: https://www.cbioportal.org (accessed on 4 January 2022).
